# Supplementary figures and images for: Gibberellic acid-mediated transcriptional divergence underlies cold stress adaptation in two diploid cotton species
Source: PeerJ. 2025 Jul 3;13:e19721. doi: 10.7717/peerj.19721 (PMC12229150; doi:10.7717/peerj.19721)

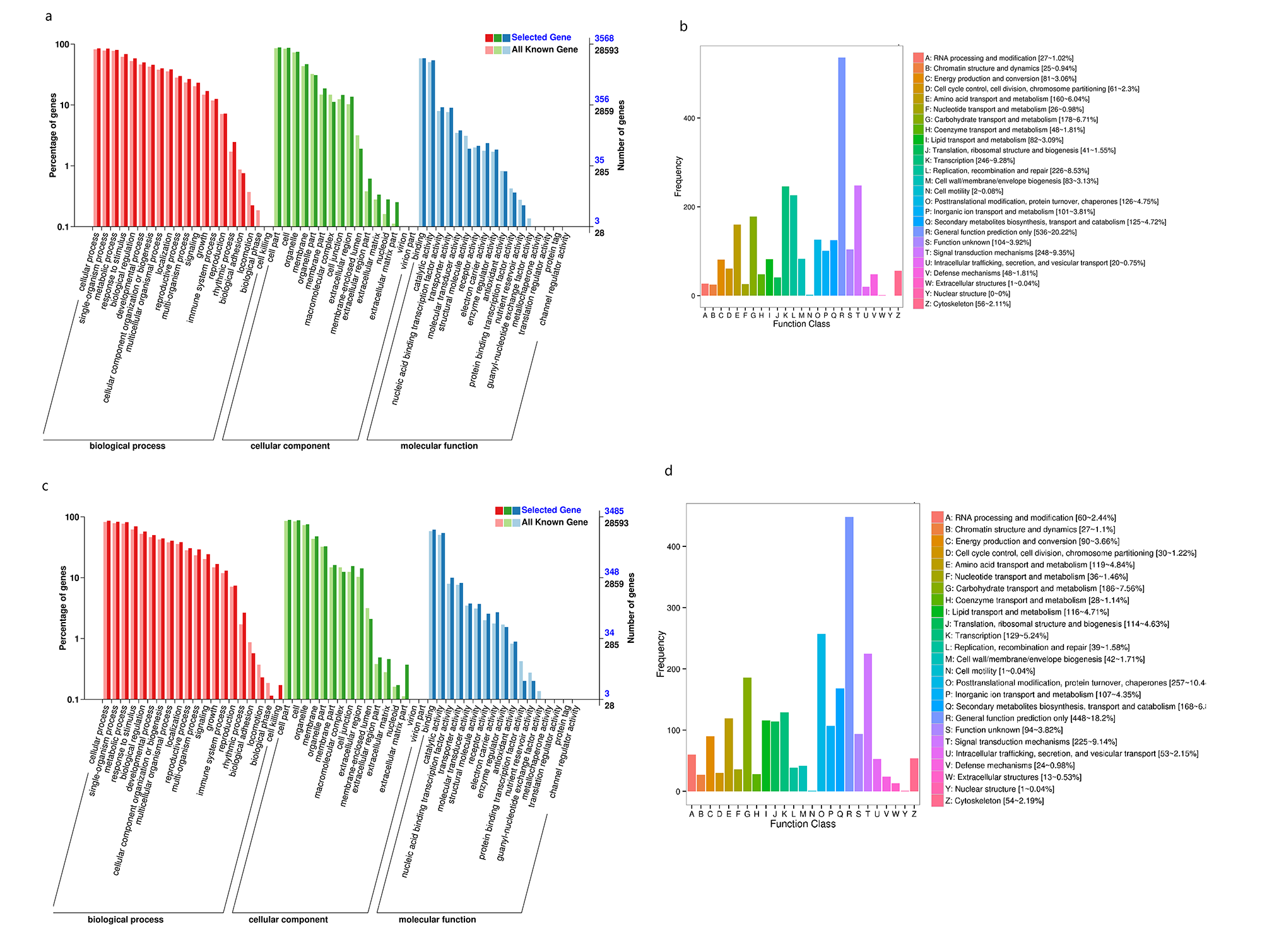

Supplement: Supplemental Information 1 [file peerj-13-19721-s001.zip › figure S1.png]

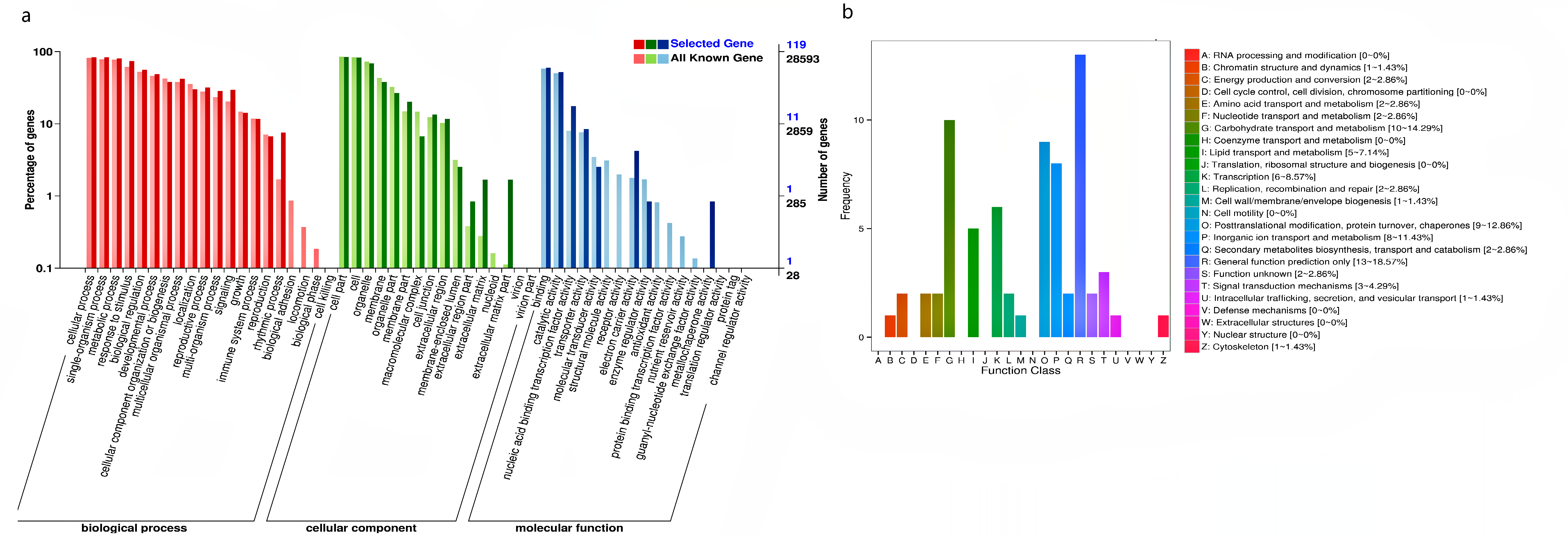

Supplement: Supplemental Information 1 [file peerj-13-19721-s001.zip › Figure S2.png]
